# Membranoproliferative Glomerulonephritis in Patients with Chronic Venous Catheters: A Case Report and Literature Review

**DOI:** 10.1155/2014/159370

**Published:** 2014-01-30

**Authors:** John Sy, Cynthia C. Nast, Phuong-Thu T. Pham, Phuong-Chi T. Pham

**Affiliations:** ^1^Division of Nephrology and Hypertension, Department of Internal Medicine, UCLA-Olive View Medical Center, 14445 Olive View Drive, 2B-182, Sylmar, CA 91342, USA; ^2^Cedars Sinai Medical Center, Department of Pathology, Los Angeles, CA 90048, USA; ^3^David Geffen School of Medicine at UCLA, Kidney and Pancreas Transplant Program, Los Angeles, CA 90095, USA

## Abstract

Chronic indwelling catheters have been reported to be associated with membranoproliferative glomerulonephritis (MPGN) *via* the activation of the classical complement pathway in association with bacterial infections such as coagulase negative staphylococcus. We herein provide supporting evidence for the direct causal relationship between chronic catheter infections and MPGN *via* a case of recurrent MPGN associated with recurrent catheter infections used for total parenteral nutrition (TPN) in a man with short gut syndrome. We also present a literature review of similar cases and identify common clinical manifestations that may serve to aid clinicians in the early identification of MPGN associated with infected central venous catheterization or *vice versa*. The importance of routine monitoring of kidney function and urinalysis among patients with chronic central venous catheterization is highlighted as kidney injury may *herald* or coincide with overtly infected chronic indwelling central venous catheters.

## 1. Introduction

Membranoproliferative glomerulonephritis (MPGN) is a pattern of disease characterized by the deposition of immunoglobulins, complement factors, or both along capillary walls and within the glomerular mesangium. The classic finding of lobular accentuation of glomerular tufts on light microscopy is attributed to mesangial hypercellularity, endocapillary proliferation, and capillary wall remodeling resulting in the formation of “double contours.” Depositions of the third component of complement (C3) with or without immunoglobulins may be observed on immunofluorescent studies [1]. The underlying etiologies of MPGN comprise a spectrum of conditions including infection, monoclonal gammopathy, autoimmune or rheumatologic disease, and dysregulation of the alternative complement pathway. It is well known that chronic infection from indwelling ventriculosystemic shunts can cause “shunt nephritis”, an entity first reported in 1965 by Black et al. after the placement of a ventriculoatrial shunt for the relief of hydrocephalus in two pediatric patients [2, 3]. Further experiments in animal studies have similarly shown a relation between chronic infections associated with indwelling catheters and MPGN [4, 5]. Although uncommon, there have been few reports of MPGN associated with central venous catheters placed for total parenteral nutrition (TPN) [6]. We herein report a case of recurrent MPGN in association with recurrent coagulase negative *Staphylococcus epidermidis* Hickman catheter infection, and review the literature for common clinical presentations of MPGN in patients requiring chronic central venous catheter placement.

## 2. Case Report

### 2.1. Clinical History and Initial Laboratory Data

A 23-year-old male with prior multiple gunshot wounds to the abdomen requiring complete small bowel resection and chronic TPN support *via* a Hickman catheter since the age of 17 presented with anasarca and low grade fevers in June 1996. Basic urine evaluations revealed 2+ blood without evidence of casts and 2.0 g proteinuria from a 24-hour collection. A serum chemistry panel revealed creatinine of 1.9 mg/dL (estimated glomerular filtration rate of 50 mL/min/1.73 m^2^), blood urea nitrogen (BUN) of 37 mg/dL, and albumin of 2 gm/dL. His baseline creatinine levels were unknown. Routine serology evaluation including human immunodeficiency virus (HIV), rapid plasma reagin (RPR), antinuclear antibody (ANA), and hepatitis B and C screen were all negative. Complement studies revealed C3 of 68 mg/dL (reference range, 90–180 mg/dL), fourth component of complement (C4) of 19 mg/dL (reference range 16–47 mg/dL), and total complement levels (CH50) of <28 mg/dL (reference range 60–90 mg/dL). Echocardiogram showed no vegetations. Blood cultures were positive for coagulase negative *staphylococcus*. Kidney ultrasound revealed right kidney measuring 10.6 cm and left kidney measuring 10.8 cm without structural abnormalities or evidence of obstruction. A kidney biopsy was performed.

### 2.2. Kidney Biopsy (June 1996)

**Figure 1:**
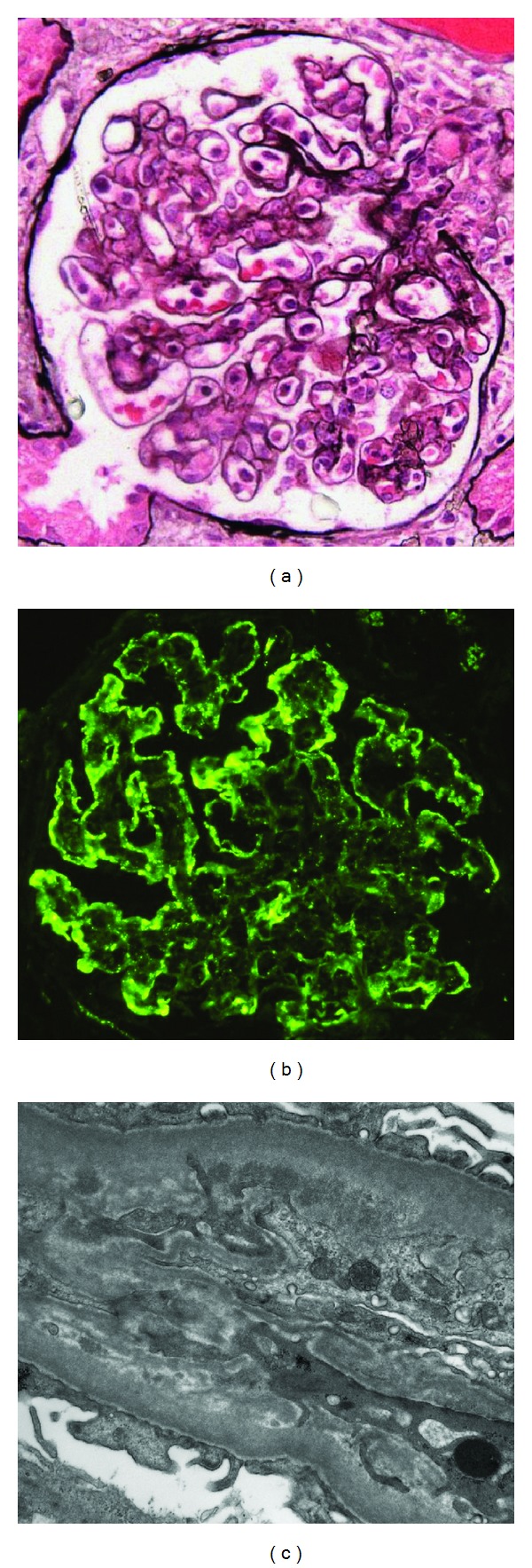
Glomerular renal biopsy findings. (a) Mesangial and endocapillary hypercellularity with a lobular pattern and segmental capillary double contours (periodic acid methenamine silver ×400). (b) Peripheral granular staining for C3 (×400). (c) Capillary wall subendothelial electron dense deposits with peripheral mesangial migration and new subendothelial basement membrane material forming a double contour (×19,000).

Light microscopy revealed 15 glomeruli showing a lobular pattern with mesangial hypercellularity, a moderate number of capillary wall double contours, and leukocytes within capillary lumina. Three glomeruli had segmental crescents. There were interstitial inflammation and edema, associated with acute tubular cell injury. Immunofluorescence microscopy disclosed five glomeruli staining for IgM (3+), C3 (3+), and kappa (trace to 1+) and lambda (trace) light chains along capillary walls in a granular pattern and peripheral distribution. Mesangial regions were stained for IgM (1 to 2+), C1q (trace), C3 (1+), and kappa (trace) and lambda (trace) light chains in a granular pattern. Electron microscopy of three glomeruli revealed small subendothelial and few mesangial electron dense deposits. There were no tubuloreticular structures in the cytoplasm of any cells (Figure 1).

Diagnoses of membranoproliferative glomerulonephritis type I and acute tubulointerstitial nephritis were rendered.

### 2.3. Clinical Follow-Up

The patient received vancomycin and underwent Hickman catheter replacement with subsequent rapid and complete resolution of his acute kidney injury (serum creatinine improved to 1.3 mg/dL), proteinuria, and anasarca. Due to the temporal association of treatment and renal disease resolution, the MPGN was presumed to be secondary to *staphylococcal* bacteremia.

**Figure 2:**
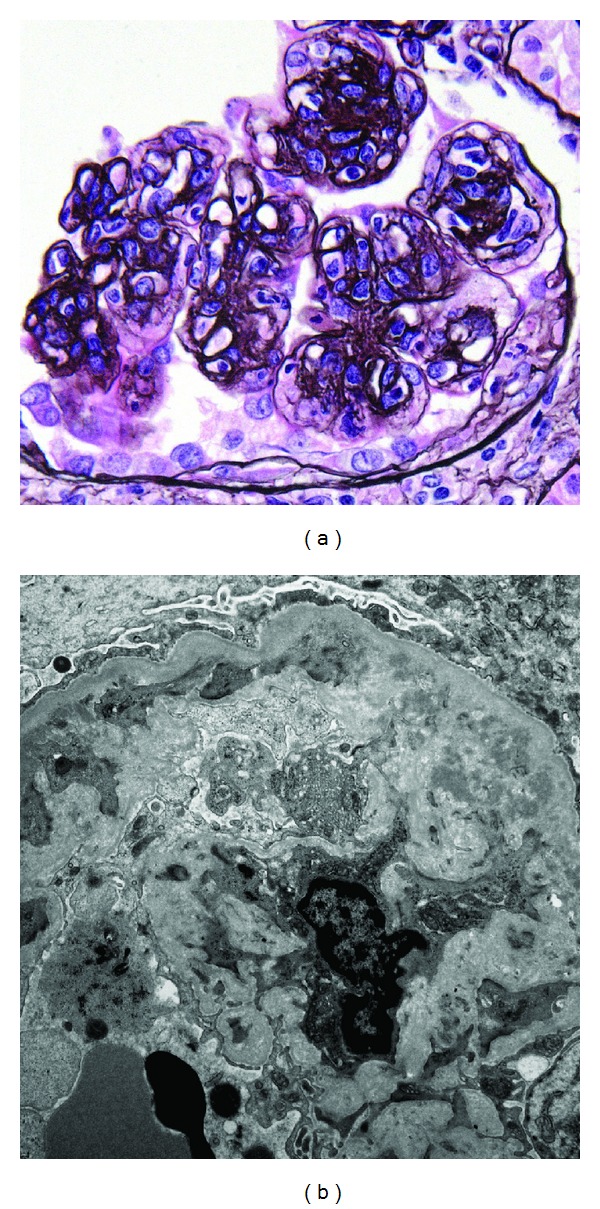
Glomerular features of second renal biopsy. (a) Lobular hypercellular glomerulus with capillary wall double contours (periodic acid methenamine silver ×600). (b) Capillary wall with subendothelial deposits and peripheral mesangial migration and interposition producing a double contour (×7200).

He was lost to follow-up for several years until February 2010 when he presented with upper extremity edema and chills. On admission he had anemia, reduced kidney function, and hypoalbuminemia. Again, he was found to be infected with coagulase negative staphylococcus bacteremia (*S. epidermidis*). His spot urine protein to creatinine ratio at presentation was 2.4 g/g creatinine but increased to 5.8 g/g over several days without associated blood pressure changes. Routine laboratory investigations revealed creatinine of 2.2 mg/dL, BUN 19 mg/dL, WBC 4,400/mm^3^, and hemoglobin 5.9 g/dL. Urinalysis revealed 300 mg/dL protein, large blood, large leukocyte esterase, 196 WBC/high power field (HPF), 224 RBC/HPF, 33 hyaline casts, few WBC clumps, 14 granular casts, and 24 cellular casts. He was treated with vancomycin pending repeat evaluation of the underlying nephrotic syndrome. Of interest, the patient commented that “every time I swell up, they give me antibiotics and the swelling goes away.” Further evaluation of his renal disease was again pursued as all his previous medical records were lost in a hospital fire. Serum protein electrophoresis (SPEP), urine protein electrophoresis (UPEP), serum protein immunofixation (SPIF), urine protein immunofixation (UPIF), antineutrophil cytoplasmic antibody (ANCA), RPR, ANA, and HIV were all negative. C3 was low at 71 mg/dL with normal C4 of 23 mg/dL. A kidney ultrasound revealed normal sized kidneys (right 11.0 cm and left 11.7 cm) without structural abnormalities. Evaluation for subacute bacterial endocarditis was negative. His Hickman catheter was replaced and subsequent blood cultures confirmed resolution of his bacteremia. His proteinuria improved markedly (greater than 50% reduction) within a few days of antibiotic therapy initiation. Serial creatinine measurements documented improvement in his creatinine to 1.75 mg/dL within 20 days of presentation. A repeat kidney biopsy performed in June 2010 confirmed MPGN type I (Figure 2), with acute tubulointerstitial nephritis and mild-to-moderate chronic renal parenchymal injury.

In July 2010, he presented for the third time with anasarca, fevers, and acute kidney injury (creatinine of 3.29 mg/dL, elevated from baseline level 1.7–2.0 mg/dL). Initial urinalysis showed 100 mg/dL protein, large blood, 215 WBC/HPF, 252 RBC/HPF, and 42 hyaline casts. Complement levels revealed C3 of 45 mg/dL, C4 of 18 mg/dL, and CH50 < 13 mg/dL. Blood cultures revealed coagulase negative staphylococcus bacteremia (*S. epidermidis*) and spot urine protein-creatinine ratios rapidly rose from 5.1 g/g to a peak of 9.5 g/g within 2 days without accompanying blood pressure changes. Treatment with vancomycin and Hickman line replacement led to rapid reduction in proteinuria and anasarca with creatinine improving to his recent baseline of 1.7 mg/dL by October 2010. As the two previous kidney biopsies demonstrated MPGN type I, this third episode of acute kidney injury accompanied by hematuria and nephrotic range proteinuria was attributed to a recurrence of MPGN secondary to recurrent Hickman catheter infection.

## 3. Discussion

The pathophysiology of MPGN caused by chronic indwelling central catheters has been previously described [4, 6, 8]. The production of immunoglobulins against an infectious agent and the subsequent binding of two or more of these immunoglobulins result in activation of C1, which then cleaves C4 and C2 to generate C4b and C2a to form C4b2a, the classical pathway convertase, leading to the activation of C3 convertase and generation of the terminal complement complex. Glomerular involvement is instigated by deposition of immune-complexes and complement factors of the classical and terminal pathway in the subendothelial region of capillary walls [6]. The injury phase of MPGN is characterized by the influx of leukocytes with associated cytokine and protease release, inducing capillary wall damage and ensuing hematuria and proteinuria. In addition to *Staphylococcus*, other bacteria reported in association with MPGN include *Mycobacterium tuberculosis*, *streptococci*, *Propionibacterium acnes*, *Mycoplasma pneumoniae*, *brucella*, *Coxiella burnetii*, *nocardia*, and *Meningococcus* [8].

A literature search for biopsy proven MPGN associated with chronic central venous catheterization revealed only three cases [6, 7]. In these three reported cases, the central venous catheter was used for home parenteral nutrition for short bowel syndrome (Table 1). Of note, all patients had multiple (five to seven) episodes of infectious catheter complications prior to overt renal manifestations. Specific renal presentations ranged from incidental finding of microscopic hematuria, mild proteinuria (0.3 g/g creatinine), and granular casts in one patient to an insidious or relatively rapid rise in serum creatinine over 18 days to 2 months in the two other patients. Patients with increasing serum creatinine had concurrent significant proteinuria and an active urinary sediment including microscopic hematuria with or without cellular casts. Associated extrarenal clinical manifestations reported include edema/anasarca, fevers, and/or palpable purpura due to biopsy proven leukocytoclastic vasculitis. Complement levels varied from normal to significantly depressed. Blood and catheter tip cultures obtained in three out of four cases revealed *Staphylococcus epidermidis*. Following catheter replacement and appropriate antibiotic administration, all patients promptly and markedly improved in renal function, proteinuria, and/or hematuria. Of interest, signs of recovery such as reduction in proteinuria may be noted within a few days and fall in serum creatinine within 1-2 weeks of antibiotic therapy and catheter removal (8, present case). Complete recovery of renal function may occur within three to 10 months. Unfortunately, in our current case, only partial renal functional recovery and reduction in proteinuria were observed following the third documented episode of infection with glomerulonephritis. This likely was due to significant renal parenchymal scarring consequent to inadequate treatment of prior infectious insults in association with poor patient compliance and follow-up.

Recurrent biopsy proven MPGN in parallel with recurrent line infection/bacteremia, as observed in the current case, leaves little doubt regarding a direct causal relationship between chronic central line infections and MPGN. Subtle renal manifestations such as microscopic hematuria and slow rise in serum creatinine may herald overtly apparent manifestations of catheter infections; therefore, routine urinalysis and close monitoring of serum creatinine in patients with chronic central venous catheterization are indicated to allow early detection of catheter infection and prevention of progressive kidney injury. It should be noted that animal studies involving sheep and baboons have shown that renal parenchymal injury may occur even prior to overt renal manifestations [4, 5]. Nevertheless, some extent of disease reversal is expected with prompt intervention, including appropriate antimicrobial therapy and removal or replacement of the indwelling catheter, as reported [6, 7].

In summary, we have presented a case of recurrent MPGN associated with recurrent bacteremia from a chronically indwelling Hickman catheter in an adult. Given the repeated strong temporal correlations of MPGN with recurrent catheter infections, the latter is likely the key factor in the development of MPGN in this setting. In a patient with an infected catheter and concurrent evidence of kidney injury, with or without a prior biopsy-proven MPGN, short time allowance for antibiotic treatment and catheter replacement may be indicated prior to renal biopsy performance, particularly when the only abnormal serologic finding is a low C3 level. However, it should be emphasized that a kidney biopsy should be considered if renal function and/or proteinuria do not resolve within 2–4 weeks, or if serologic testing suggests the possibility of another disease entity.

In conclusion, patients with chronic indwelling central venous catheters should be given routine surveillance for both infections and markers of kidney injury including serum creatinine and urinalysis. Similarly, patients should be educated to recognize early signs and symptoms of infections as well as development of unusual urinary foaming and/or change in urine output and color.

## Figures and Tables

**Table 1:** Clinical manifestations of reported cases and current case.

References	Medical history	Initial presentation	Baseline creatinine (mg/dL)	Presenting creatinine (mg/dL)	Urinalysis	Complements (mg/dL)	Renal Biopsy	Blood cultures	Number of catheter changes
Yared et al. [7]	66-year-old male with mesenteric ischemia and bowel resection with parenteral nutrition for hyperalimentation	Worsening kidney function and new skin rash	1.5	3.2	>25 RBC/HPF, proteinuria, 4–10 granular casts	“Normal” complements (values not reported)	MPGN	*S. epidermidis* and *C. jeikeium *	6

Yared et al. [7]	45-year-old female TAH/BSO complicated by ischemic bowel requiring resection, required parenteral nutrition for hyperalimentation	Worsening kidney function, new skin rash, and severe anemia	1.8	7.7	Proteinuria and hematuria with RBC and mixed-cell casts	Initially normal complements, then C3 and C4 levels slightly depressed	MPGN	Unknown	5

Ohara et al. [6]	13-year-old male midgut volvulus and resection of necrotic ileum, required parenteral nutrition for hyperalimentation	Hematuria and proteinuria on routine urinary screening	Unknown	0.6	Many RBCs, 10–15 WBC, 1-2 granular casts/HPF	C3 30 (low), C4 8 (low), CH50 < 10 (low)	MPGN	*S. epidermidis *	7

Current case report	23-year-old male multiple gunshot wounds to abdomen at age 17, required parenteral nutrition for hyperalimentation	First episode July 1996: proteinuria, hematuria, and renal insufficiency on routine testing	Unknown	1.9	2+ blood, >100 RBC, no cellular casts	C3 69 (low), C4 19 (low-normal), CH50 < 28 (low)	MPGN	*S. epidermidis *	Unknown
		Second episode February 2010 at age 37: fevers, anasarca, and renal insufficiency	1.3–1.5	2.2	Protein 300 mg/dL, large blood, WBC 196, RBC 224, +hyaline, granular, and cellular casts/HPF	C3 71 (low), C4 23 (low-normal), CH50 < 13 (low)	MPGN (biopsy done June 2010)	*S. epidermidis *	>2
		Third episode July 2010: anasarca and fatigue	1.7–2.0	3.3	Protein 100 mg/dL, large blood, WBC 215, RBC 252, 42 hyaline casts/HPF	C3 45 (low), C4 18 (low-normal), CH50 < 13 (low)	No biopsy*	*S. epidermidis *	>2

Abbreviations: TAH/BSO: total abdominal hysterectomy and bilateral salpingooopherectomies; *S. epidermidis*: *Staphylococcus epidermidis*; *C. jeikeium*: *Clostridium jeikeium*; RBC: red blood cells; WBC: white blood cells; HPF: high power field.

*Presumptive diagnosis of recurrent MPGN based on previous biopsy findings, clinical course, and response to appropriate therapy.
